# An examination of referrals declined for chronic pain care: There is increasing mental health complexity within care-seeking patients with chronic pain over time

**DOI:** 10.1080/24740527.2024.2337074

**Published:** 2024-04-02

**Authors:** Rachael Bosma, Brittany N. Rosenbloom, Emeralda Burke, Christian Aquino, Cara Stanley, Kimberly Coombs, Adriano Nella, Shamalla James, Hance Clarke, David Flamer, Anuj Bhatia, John Flannery, Andrew Smith, Tania Di Renna

**Affiliations:** a Toronto Academic Pain Medicine Institute, Women’s College Hospital, Toronto, Ontario, Canada; b Department of Anaesthesia and Pain Management, Toronto General Hospital, Toronto, Ontario, Canada; c Department of Anaesthesia and Pain Management, Transitional Pain Service, Toronto, Ontario, Canada; d Pain Management Centre, Sinai Health, Toronto, Ontario, Canada; e Comprehensive Integrated Pain Program – Interventional Pain Service, Toronto Western Hospital, Toronto, Ontario, Canada; f Department of Anesthesia and Pain Medicine, University of Toronto, Toronto, Ontario, Canada; g Institute of Health Policy, Management, and Evaluation, University of Toronto, Toronto, Ontario, Canada; h Comprehensive Integrated Pain Program, Toronto Rehabilitation Institute, Toronto, Ontario, Canada; i IPARC, Center for Addiction and Mental Health (CAMH), Toronto, Ontario, Canada

**Keywords:** Chronic pain, mental health, health care referral

## Abstract

**Background:**

Chronic pain is a complex disease that requires interprofessional care for effective management. Despite the need for multidisciplinary care, disease and health care inequities can prevent individuals from attaining adequate treatment. Factors such as mental health, cost, and distance to a health care center can contribute to health care accessibility inequality. The aim of this study is to examine declined referrals at the Toronto Academic Pain Medicine Institute (TAPMI) to determine the reason for declining care and number of declined referrals.

**Methods:**

A retrospective chart review of all declined referrals at TAPMI in 2018 and 2022 was conducted. Referral documentation and the intake decision were extracted from the electronic medical charts by the research team and verified by the clinical intake team. Chi-square tests were conducted to determine whether the proportion of declined referrals changed between the years reviewed.

**Results:**

The number of declined referrals due to mental health complexities increased significantly from 51 (11%) in 2018 to 180 (18%) in 2022 (χ^2^ = 10.9, *P* = 0.0009). A significant rise in the number of declines due to mental health service requests was also observed (χ^2^ = 24.53, *P* < 0.00001). Other common reasons for declined referrals in 2018 and 2022 included duplicate service, no primary care provider, and health care service changes.

**Conclusion:**

Mental health complexities continue to be a significant barrier to health care service acquisition for individuals living with chronic pain. The increase in patient complexity from 2018 to 2022 highlights the need for integrated health care resources.

## RÉSUMÉ

**Contexte**: La douleur chronique est une maladie complexe qui nécessite des soins interprofessionnels pour une prise en charge efficace. Malgré la nécessité de soins multidisciplinaires, les maladies et les iniquités en matière de soins de santé peuvent empêcher les individus d’obtenir un traitement adéquat. Des facteurs tels que la santé mentale, le coût et la distance d’un centre de soins de santé peuvent contribuer aux inégalités en matière d’accessibilité des soins de santé. Le but de cette étude est d’examiner les demandes de consultation refusées à l’Institut universitaire de médecine de la douleur de Toronto (TAPMI) afin de déterminer la raison du refus des soins et le nombre de demandes de consultation refusées.

**Méthodes**: Un examen rétrospectif de toutes les demandes de consultation refusées au TAPMI en 2018 et 2022 a été effectué. La documentation relative à la demande de consultation et la décision d’admission ont été extraites du dossier médical électronique par l’équipe de recherche et vérifiées par l’équipe d’admission clinique. Des tests de chi-carré ont été effectués pour déterminer si la proportion de demandes de consultation refusées avait changé entre les années examinées.

**Résultats**: Le nombre de demandes de consultation refusées en raison des complexités de la santé mentale a considérablement augmenté, passant de 51 (11 %) en 2018 à 180 (18%) en 2022 (χ2 = 10,9, P = 0,0009). Une augmentation significative du nombre de refus attribuables aux demandes de services de santé mentale a également été observée (χ2 = 24,53, P < 0,00001). Parmi les autres raisons courantes pour lesquelles les demandes de consultation ont été refusées en 2018 et 2022, on compte la duplication du service, l’absence de prestataire de soins primaires et les changements dans les services de soins de santé.

**Conclusion**: Les complexités de la santé mentale continuent d’être un obstacle important à l’acquisition de services de soins de santé pour les personnes vivant avec une douleur chronique. L’augmentation de la complexité des patients de 2018 à 2022 souligne la nécessité de ressources de soins de santé intégrées.

## Introduction

Chronic pain affects approximately 19% of the Canadian adult population and is one of the leading contributors to years lived with disability globally.^1,2^ The Canadian government estimates that the prevalence of chronic pain will increase by 17.5% from 2019 to 2030, further necessitating the development of comprehensive health care options.^3^ The complexity of chronic pain requires an interprofessional treatment approach utilizing the biopsychosocial model to manage the patient specific factors that may play a role in pain perception and in response to pain-relieving treatments.^4^

Despite the high prevalence of pain and the necessity of interprofessional care approaches, access to comprehensive pain care is a challenge for many seeking care because these services are often sparse, have long wait times, and are often located in urban centers.^5^ Access to interprofessional care is further limited by stringent exclusion criteria within interprofessional clinics.^6^ For example, approximately one in four Canadian multidisciplinary pain treatment centers excluded patients if a co-occurring mental health disorder and/or a substance use disorder was present.^6^ The Canadian Pain Task Force has recently acknowledged the disparity in health care due to mental health concerns and has highlighted equitable access to comprehensive care as a pillar of focus.^3^

Among the various biological, psychological, and social factors that can interfere with pain management, mental health–related issues are among the most prominent and well established. Mental health–related complexities include mood, anxiety, stress, personality, substance use, and psychotic disorders.^7^ Mental health disorders have been found to be more prevalent in the chronic pain population when compared to the general population, although the specific prevalence varies depending on the diagnosis.^8^ For example, a recent meta-analysis of the prevalence of co-occurring chronic pain and posttraumatic stress disorder revealed that among persons with pain the rate is 11.7%; however, in general populations it is 5.1%.^9^ The higher pain intensity, increased disability, and reduced quality of life that are often associated with the co-occurrence of a mental health issue and chronic pain further complicate pain management recommendations.^10–12^ Despite this, individuals living with complex mental health issues lack access to care due to common exclusion criteria around mental comorbidities for accessing pain clinics. However, the impact of co-occurring mental health on access to care for the population with chronic pain remains unknown.

The pandemic exacerbated the systemic inequalities perceived by people living with chronic pain in Canada.^13^ It also led to a rise in the demand for mental health services. Given the common comorbidities of pain and mental health needs, the lack of integrated pain and mental health services in many interprofessional pain clinics, and the rise in the demand for pain services, it is essential to understand the reasons people are being declined chronic pain services, who is impacted, and whether this has shifted pre versus post pandemic.

To address this, we conducted a retrospective review of all declined referrals at the Toronto Academic Pain Medicine Institute, a partnership of five Toronto academic pain institutions that receive approximately 7000 referrals every year. We describe and contrast the reason and number of declined referrals in a prepandemic (2018) and postpandemic (2022) year.

## Methods

This retrospective chart review was conducted at the Toronto Academic Pain Medicine Institute at Women’s College Hospital and received approval from the Assessment Process for Quality Improvement Projects ethics board (APQIP No. 2023–0007-P). The Toronto Academic Pain Medicine Institute (TAPMI) is a comprehensive, interdisciplinary pain program supported by a collaborative partnership across five institutions in Toronto, offering a single-site entry system for referrals and a centralized referral assignment process. The TAPMI clinic receives between 3500 and 7000 referrals per year. Two trained members of the research team (social work student S.J. and research coordinator E.B.) reviewed the referral documentation for all declined referrals in 2018 and 2022 to determine the reason for the referral decline. These years were chosen because TAPMI was well established by 2018 and 2022 represents a return to normalcy from COVID-19. If the reason for declining the referral was not clear from the documentation in the patient’s electronic medical chart, the research team reviewed the referral package with a registered practical nurse on the intake team. Some referrals contained multiple reasons for the decline, in which case all reasons were reported.

We took a modified content analysis approach to categorizing the themes of the declined referrals.^14^ Free text was used to document referral information, which was used to create codes for categorization of the reasons referrals were declined. The research team (R.B., A.N., E.B., B.R.), in collaboration with the intake team (T.D., C.S., C.A., K.C.), continually refined the code list to best categorize the data. Once the preliminary data were pulled from the charts and coded, the codes were grouped into categories for further analysis, which led to broader categories (i.e., themes). Rigorous discussions with the research and intake nursing team based on inductive reasoning and prior literature led to the development of the categories shown below. For example, “unresolved mental health service needs” includes referrals that were declined for reasons of untreated and unstable mental health (e.g., untreated and active schizophrenia, not seeking treatment for substance use disorder), those who declined services for mental health care (e.g., refused treatment), and those not actively receiving mental health care despite the need for this care (e.g., suspected schizophrenia but declined participating in an assessment). Note that referrals were not declined due to the presence of any mental health diagnosis (e.g., generalized anxiety disorder or substance use disorder), but exclusion criteria were determined by clinical decision making per case on the basis of unstable and untreated mental health.

### Statistical Methods

To determine whether the proportion of declined referrals differed between 2018 and 2022, chi-square tests were conducted using SPSS v29.^15^ For all tests a *P* value of <0.05 was considered significant.

## Results

**Figure 1.**
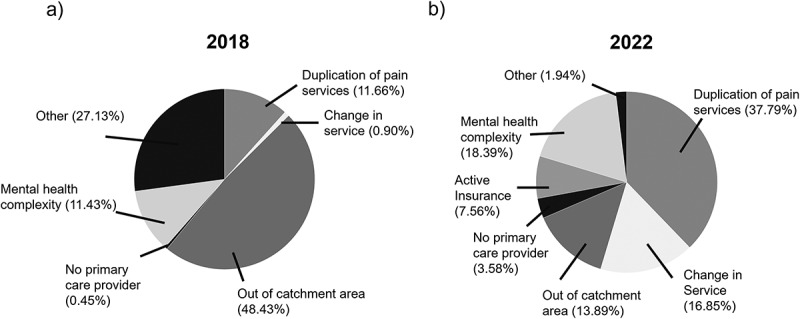
The most prominent reason for declined referrals in 2018 and 2022. The top reasons for declined referrals in (a) 2018 and (b) 2022. The other category includes various declined referrals such as incomplete referrals, inappropriate referrals (not related to a chronic pain condition), or requests for pain services not provided.

A total of 3520 referrals were received at TAPMI in 2018, with 446 being declined (12.67%). In 2022, 6796 referrals were assessed by the intake team and 979 were declined (14.41%). The top reasons for declined referrals in 2018 and 2022 are depicted in Figure 1. In 2018, 278 of the declined referrals were among females (62.33%) and 168 were among males (37.67%), with an average age of 53.71 (SD = 14.84) years. In 2022, 685 of the declined referrals were among females (69.97%) and 294 were among males (30.03%), with average age of 52.08 (SD = 17.05) years.

### Mental Health

In 2018, a total of 51 referrals (11.43% of declined referrals) were declined due to co-occurring complexity in mental health concerns (e.g., untreated substance use disorder and instability due to borderline personality disorder) that precluded pain care in 2018. In 2022 the number of referrals that were declined due to a complexity in mental health that precluded pain care significantly increased to 180 referrals (18.39%; χ^2^ = 10.9, *P* = 0.000962). Upon further investigation, the proportion of those with complex mental health diagnoses did not change over time (χ^2^ = 0.063, *P* = 0.80) but the proportion of those seeking services for mental health services did significantly increase (χ^2^ = 24.53, *P* < 0.00001; Table 1).

**Table 1.** The number of referrals declined in 2018 and 2022 due to mental health complexities.

Reason for declined referral		2017/2018, *n* (%)	2022/2023, *n* (%)
Total mental health complexities		51 (11.43)	180 (18.39)
Mental health service		10 (2.24)	94 (9.60)
	Requesting one-on-one counseling/therapy	6 (1.35)	0 (0)
	Requesting one-on-one psychiatric care	3 (0.67)	72 (7.35)
	Unresolved mental health service needs	1 (0.22)	22 (2.45)
Mental health concerns		41 (9.19)	86 (9.90)
	Mental health not specified	0 (0)	10 (1.02)
	Substance use: not seeking treatment	10 (2.24)	20 (2.04)
	Schizophrenia	0 (0)	2 (0.20)
	Personality disorders	19 (4.26)	17 (1.74)
	Complex trauma presentations	12 (2.69)	37 (3.78)

### Duplication of Pain Services

**Figure 2.**
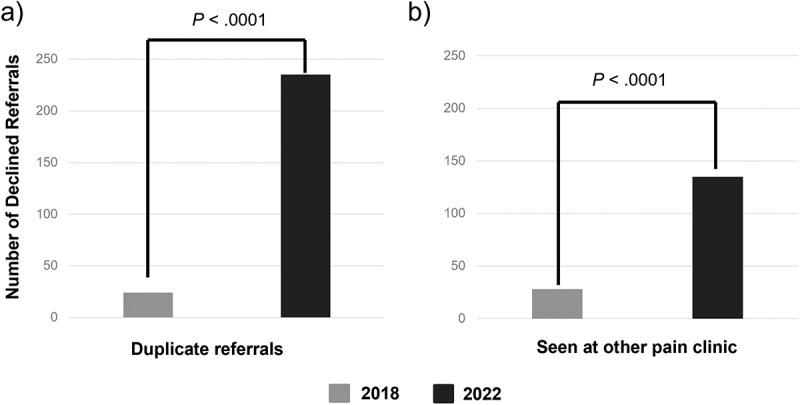
The number of referrals that were declined in 2018 and 2022 due to duplicate care: (a) duplicate referrals and (b) the patient was seen at another pain clinic within the past year.

Referrals requesting duplicate services, which include multiple referrals for the same patient and patients who received the same care offered at another pain clinic in the previous year, are shown in Figure 2. Duplicate referrals increased significantly from 24 (5.38%) to 235 (24.00%) between 2018 and 2022 (χ^2^ = 71.45, *P* < 0.0001). The number of referrals that were declined due to receiving care at a separate pain clinic within the previous year increased significantly from 28 referrals in 2018 (6.28%) to 135 referrals in 2022 (13.79%; χ^2^ = 17.07, *P* < 0.0001).

### No Primary Care Provider

In 2018 only 2 referrals were declined because they did not have a primary care provider (0.45%). This number significantly increased to 35 declined referrals in 2022 (3.58%; χ^2^ = 11.84, *P* = 0.00058).

### Change in Clinic Service Criteria

Between 2018 and 2022 there was a change in the number of declined referrals due to changes to the services offered at the clinic, which include referrals for services that have been paused (e.g., ketamine infusions), active insurance claims (Workplace Safety and Insurance Board claim), or if the patient is outside of the TAPMI catchment area. The number of patients who were declined due to being outside of the catchment area decreased from 216 (48.43%) to 136 (13.89%). The number of patients who were declined due to service pause of clinical programs that were once accepting referrals increased from 4 in 2018 (0.90%) to 165 in 2022 (16.85%). In 2018 zero referrals were declined due to an active insurance claim, whereas 74 referrals were declined in 2022 (7.56%).

## Discussion

We set out to explore the barriers to chronic pain care, determine the number of patients declined due to exclusion criteria used by TAPMI partner sites, and explore the changes in declined referrals between 2018 and 2022. We found that common reasons for declining patient referrals were due to the complexity in mental health, lack of a primary care provider, duplicate care requests, and service-related changes.

Our results indicate that the co-occurrence of complex mental health issues, in addition to a lack of services required to manage mental health complications, is a significant barrier to care for individuals living with chronic pain. Consistent with the marked rise in mental health distress observed during the COVID-19 pandemic,^13,16^ we found a significant increase from 11% in 2018 (prepandemic) to almost 20% in 2022 (postpandemic) in the number of individuals seeking pain care alongside complexities in mental health. Despite the Canadian Pain Task Force acknowledging that the most impactful method to treat chronic pain is through the application of the biopsychosocial model and a multidisciplinary approach, current models of care lack the resources or funding to effectively integrate mental health care.^3^ With approximately one in four Canadian multidisciplinary pain treatment centers excluding patients with a co-occurring mental health and/or substance use disorder, access to care is challenging.^6^ The disproportionate rise in referrals requesting one-on-one therapy or consultation with a psychiatrist indicates the necessity of integrated mental health and pain management. However, even with a psychologist or psychiatrist present, multidisciplinary pain centers remained apprehensive to include patients with complex mental health needs.^6^ Psychologists who specialize in pain management are rare, with a majority of psychologists expressing low perceived competency and confidence to manage pain.^17^ Despite the high interest in pain education, pathways to equip psychologists with pain management skills are not yet widespread.^17^ Furthermore, to address overwhelming patient volumes and long wait times, many interprofessional chronic pain clinics offer group-based programming, which may limit the enrollment for individuals with co-occurring mental health. Literature suggests that enrolling individuals into group management sessions without managing psychiatric conditions could disrupt care for other members and interfere with treatment outcomes.^18,19^ To tackle the complexity of the patient population, further specialized resources that combine both pain and mental health care are required. Additionally, patients with co-occurring pain and mental health concerns may require longitudinal care. This is at odds with health care models, such as in Ontario, in which funding for pain care is based on the number of new referrals and patients are expected to be discharged within a year (i.e., acute care model).

Duplicate services, which includes duplicate referrals and referrals for services already provided, were a common reason for declined referrals. We observed a substantial increase in the number of duplicate referrals, which rose by approximately five times from 24 declined referrals in 2018 (5.38%) to 235 declined referrals in 2022 (24.00%). This could be indicative of patients facing diagnostic uncertainty and seeking care from multiple providers who then provide the same referral to the pain clinic. Duplicate referrals could also reflect long wait times in which patients are re-referred when they have not received an appointment in a timely manner. Wait times for chronic pain care in Ontario have been estimated to be 176 days, with some conditions exceeding 1 year for treatment. With wait times of 6 months being deemed medically unacceptable and linked with poor patient outcomes, patients or health care providers may attempt a re-referral.^20^ Regardless, multiple referrals for the same patient are administratively resource intensive and contribute to inefficiencies in the triage process.

We also report a significant increase in the number of patients who were declined because they already received comparable services from other health service providers, from 6.28% in 2018 to 13.79% in 2022. Referral decisions at TAPMI are made after careful consideration of the referral documentation, patient history, and the services requested on the referral. In response, referring health care providers are offered e-consultation services to further support the patients care management plan. Despite utilizing appropriate care (e.g., physiotherapy, pain-related psychotherapy, pain medication management) in the past, these patients continue to be referred to a specialist pain service.

It is possible that the rise in both duplicate referrals and patients seeking duplicate care may indicate that patients are dissatisfied with the service they have received or that the care received does not align with patient expectations. For example, 50% of individuals living with pain reported being satisfied with the care they received.^21^ Literature also reveals that patients often set high expectations for the treatment to reduce pain (e.g., “find a cause for my pain and fix it”), which does not align with the treatment outcomes set by health care professionals, which focus on improving pain function and reducing disability.^22,23^ Patient expectations have also been shown to predict treatment outcomes, and a misalignment may predispose the patient to being dissatisfied with care.^24^ Discharge planning may also play a role in seeking duplicate care because prior literature has shown that effective discharge planning can reduce hospital readmission for chronic conditions and improve patient satisfaction, but the effect of discharge within chronic pain remains unknown.^25,26^ Toward this effort, patient–provider communication, whether from the initial referral process to set timeline expectations, the initial appointment to set expectations about care, or a separate clinic upon discharge, is helpful in adequately managing patients with chronic pain. Future studies could work toward understanding the underlying factors that may be motivating patients to seek additional chronic pain care. Such information could help alleviate the excessive use of health care resources required to manage duplicate service requests.

The lack of resources to provide consistent long-term chronic pain management from specialist tertiary care clinics necessitates that primary care providers (PCPs) help manage a treatment plan and prescriptions in collaboration with the patient after tertiary care. However, according to recent estimates from the Canadian government, more than one in five Canadians do not have a designated family practitioner, ranking Canada as 29th out of 36 high-income nations.^27^ Ontario specifically has the largest population per primary care provider in all of Canada, further straining health care providers and limiting access.^27^ The decline in access to primary care was further exacerbated during the pandemic, in which there was a marked increase the percentage of family physicians who stopped practicing.^28^ This is exemplified by the approximately 3.5% of declined referrals for individuals who do not have a PCP. Furthermore, the increase in patients who do not have a PCP is underrepresented in our sample, because mid-2022 the TAPMI clinics relaxed the requirement to have a PCP to facilitate access to chronic pain care. Not only does the decline of PCPs impact patients with chronic pain by disrupting access to a tertiary pain clinic and interfere with long-term management plans, but evidence suggests that PCPs help to lower total health care costs, improve population health, and reduce emergency visits for chronic conditions.^29,30^

Service-related changes at TAPMI, such as treatment service pauses, and catchment area changes also provide insight into the changes in widespread management of chronic pain across the province. TAPMI provides highly specialized and novel pain management programs such as management for young adults, neuromodulation, and pelvic pain physiotherapy that may not be available elsewhere. Not only are the specialized services highly sought after but, due to the lack of programs providing similar service elsewhere, the catchment area is expanded to accommodate individuals in need. The expanded catchment area to include patients from Hamilton or Thunder Bay, in combination with the abundance of referrals, results in an extended waitlist for treatment. Highly specialized services can create an imbalance in the supply of resources and the demand from patients and result in a waitlist of over 1 year for pelvic pain care. Further research is required to assess the inequality of care and guide clinicians to develop new programs that fill the health care gap. Despite the rising prevalence of chronic pain, the development of resources to accommodate the patient population are lagging.

The most notable limitation of the study is due to the nature of the retrospective design lacking data on confounding variables. Additionally, a lack of standardized reporting for the declined referrals limited our understanding of the specific reasons for the declined referral. Though this may not have impacted the overall understanding of the barriers to health care, novel and nuanced information may have been lost. Additionally, TAPMI is a continually evolving health care center that tailors care to the needs of the public based on current literature and research. As a result, services offered and referral exclusion criteria change to meet patient and system needs and may have resulted in an underestimation of results.

## Conclusion

This study provides key insight into the service operations for chronic pain and highlights barriers and inequities related to care. Mental health has been found to have acted as a barrier to comprehensive chronic pain care in 2018, which has only continued to grow as of 2022. Whether the underlying cause of the increase is due to the increased complexity of the patient population or due to the effect of the COVID-19 pandemic on mental health remains unknown. Other factors that lead to a change in the number of declined referrals include the lack of a PCP, duplicate referrals, and duplicate services. Widespread systematic health care changes which increase the ability for patients in need to receive care are desperately required to tackle the rising rate of chronic pain and lack of service providers. As patients with chronic pain become more complex and require more advanced care to treat, the further development and implementation of resources to manage this complexity is required.
